# Hepatitis B virus infection among men who have sex with men and transgender women living with or at risk for HIV: a cross sectional study in Abuja and Lagos, Nigeria

**DOI:** 10.1186/s12879-021-06368-1

**Published:** 2021-07-06

**Authors:** Olusegun A. Adeyemi, Andrew Mitchell, Ashley Shutt, Trevor A. Crowell, Nicaise Ndembi, Afoke Kokogho, Habib O. Ramadhani, Merlin L. Robb, Stefan D. Baral, Julie A. Ake, Manhattan E. Charurat, Sheila Peel, Rebecca G. Nowak, on behalf of the TRUST/RV368 study group, Manhattan Charurat, Julie Ake, Aka Abayomi, Sylvia Adebajo, Stefan Baral, Trevor Crowell, George Eluwa, Charlotte Gaydos, Afoke Kokogho, Jennifer Malia, Olumide Makanjuela, Nelson Michael, Nicaise Ndembi, Rebecca Nowak, Oluwasolape Olawore, Zahra Parker, Sheila Peel, Habib Ramadhani, Merlin Robb, Cristina Rodriguez-Hart, Eric Sanders-Buell, Sodsai Tovanabutra, Sandhya Vasan

**Affiliations:** 1 Department of Epidemiology and Public Health, University of Maryland, School of Medicine, Baltimore, MD USA; 2 Institute of Human Virology, University of Maryland, School of Medicine, Baltimore, MD USA; 3 U.S. Military HIV Research Program, Walter Reed Army Institute of Research, Silver Spring, MD USA; 4 Henry M. Jackson Foundation for the Advancement of Military Medicine, Bethesda, MD USA; 5 Institute of Human Virology Nigeria, Abuja, Nigeria; 6 U.S. Army Medical Research Directorate – Africa, Nairobi, Kenya; 7 HJF Medical Research International, Abuja, Nigeria; 8 John Hopkins Bloomberg School of Public Health, Baltimore, MD USA

**Keywords:** HBV DNA, Sexual and gender minorities, Antiretroviral therapy, Sub-Saharan Africa

## Abstract

**Background:**

Despite the development of a safe and efficacious hepatitis B vaccine in 1982, the hepatitis B virus (HBV) remains a public health burden in sub-Saharan Africa. Due to shared risk factors for virus acquisition, men who have sex with men (MSM) and transgender women (TGW) living with HIV are at increased risk of HBV. We estimated the prevalence of HBV and associated factors for MSM and TGW living with or without HIV in Nigeria.

**Methods:**

Since March 2013, TRUST/RV368 has recruited MSM and TGW in Abuja and Lagos, Nigeria using respondent driven sampling. Participants with HIV diagnosis, enrollment as of June 2015, and available plasma were selected for a cross-sectional study and retrospectively tested for hepatitis B surface antigen and HBV DNA. Logistic regression models were used to estimate odds ratios (ORs) and 95% confidence intervals (CIs) for factors associated with prevalent HBV infection.

**Results:**

A total of 717 MSM and TGW had a median age of 25 years (interquartile range [IQR]: 21–27), 5% self-reported HBV vaccination, 61% were living with HIV, 10% had prevalent HBV infection and 6% were HIV-HBV co-infected. HIV mono-infected as compared to HIV-HBV co-infected had a higher median CD4 T cell count [425 (IQR: 284–541) vs. 345 (IQR: 164–363) cells/mm^3^, *p* = 0.03] and a lower median HIV RNA viral load [4.2 (IQR: 2.3–4.9) vs. 4.7 (IQR: 3.9–5.4) log_10_copies/mL, *p* < 0.01]. The only factor independently associated with HBV was self-report of condomless sex at last anal intercourse (OR: 2.2, 95% CI: 1.3, 3.6). HIV infection was not independently associated with HBV (OR: 1.0, 95% CI: 0.7–1.6).

**Conclusion:**

HBV prevalence was moderately high but did not differ by HIV in this cohort of MSM and TGW. Recent condomless sex was associated with elevated HBV risk, reinforcing the need to increase communication and education on condom use among key populations in Nigeria. Evaluating use of concurrent HIV antiretroviral therapy with anti-HBV activity may confirm the attenuated HBV prevalence for those living with HIV.

## Background

Despite the development of a safe and efficacious hepatitis B vaccine in 1982 [1], the hepatitis B virus (HBV) remains a public health burden in sub-Saharan Africa with a reported prevalence of about 6% [2, 3]. While mortality in sub-Saharan Africa from the World Health Organization (WHO)-targeted infectious diseases (i.e. HIV, tuberculosis and malaria) is now declining, morbidity and mortality due to viral hepatitis is increasing [2]. Globally, HBV is the leading cause of end-stage liver disease (ESLD) and hepatocellular carcinoma (HCC) [4]. In 2015, HBV infections were responsible for 887,000 deaths, in addition to 257 million individuals living with chronic HBV infections worldwide [5].

In resource-limited settings, enzyme immunoassays detecting HBV surface antigen (HBsAg) are commonly used as a standard of care diagnostic test for active HBV infection. However, concerns have been raised about the use of this test for those co-infected with HBV and HIV, in whom the targeted HBV immune markers might be suppressed [6, 7]. A more sensitive diagnostic assay that quantifies HBV-DNA rather than circulating antibodies is the polymerase chain reaction (PCR)-based molecular diagnostic assay that has served as a confirmatory and treatment progress-monitoring test for active HBV [8, 9].

Due to shared risk factors for acquisition, HBV is common in persons living with HIV (PLWHs) [10]. Furthermore, men who have sex with men (MSM) are 2 to 4 times [2] and 3 to 6 times [11] more likely to be infected with HBV and HIV, respectively, as compared to reproductive aged adults [12]. HBV-HIV co-infection is associated with a 14 fold increased risk in progressing to ESLD compared to mono-infection with either virus [13] and is also associated with increased risk of hospitalization for non-liver-related causes [14, 15]. As the advent of ubiquitous combination antiretroviral therapy has improved life expectancies of MSM and transgender women (TGW) living with HIV, these individuals are now at higher risk of developing chronic complications from comorbid infections such as HBV [16, 17].

In Nigeria, prevalence studies of HBV range between 7 to 14% among reproductive aged adults [18, 19], and in Calabar, Southeast Nigeria, 69% of patients with ESLD tested positive for HBsAg [20]. HBV prevalence among PLWH range from 4 to 8% [21–23] and as high as 10 and 18% for MSM in Ibadan and Lagos, Nigeria, respectively [24]. The objective of this study was to investigate the prevalence and factors associated with PCR-diagnosed HBV among MSM and TGW living with or at risk for HIV in Nigeria. We hypothesized that HIV infections would be associated with increased odds of HBV infection in Nigerian MSM and TGW.

## Methods

### Study design and population

The TRUST/RV368 cohort study used respondent-driven sampling (RDS) to recruit MSM and TGW between March 2013 and February 2020 in Abuja and between April 2014 and May 2018 in Lagos as previously described [25]. Eligibility criteria included the following: (1) assigned male sex at birth, (2) history of insertive or receptive anal intercourse with another man in the past 12 months, (3) adulthood (aged 16 years or older in Abuja and 18 years or older in Lagos), (4) possession of a valid RDS coupon. Age inclusion criteria differed between sites because of differences in the recommendations from the instutional review boards. Exclusion critiera included those who declined HIV counseling and testing. In addition to these criteria, participants included in this cross-sectional study had an enrollment visit at the TRUST/RV368 clinics before June 2015, an HIV diagnostic result, and plasma for retrospective HBV screening. During enrollment evaluations, spaced approximately 2 weeks apart, consented participants underwent HIV counseling and testing and provided demographic, behavioral and clinical data through in-person interviews with trained staff using a standardized questionnaire. In addition, participants received physical examinations and sexually transmitted infection (STI) diagnostics [26].

### Laboratory procedures

HBsAg screening was completed using the Genescreen HBsAg 3.0 EIA assay (Bio-Rad Laboratories, Redmond, WA). To confirm the presence of HBV and to quantify DNA, we performed in vitro PCR using the Abbott RealTime HBV assay (Abbott Molecular, Des Plaines, IL). Participants who tested positive for HBsAg with detectable HBV DNA were considered HBV-infected.

Whole blood was tested for HIV using a parallel series of rapid tests (Alere Determine, Waltham, MA and Trinity biotech Uni-Gold HIV, Wicklow, Ireland). In cases of discordance, a 3rd rapid test (Chembio Diagnostics HIV-1/2 Stat Pak test, Medford, NY) was used to confirm or exclude HIV infection as outlined by the parallel testing algorithm for at-risk individuals in Nigeria [27]. For participants living with HIV, HIV RNA was quantified using the Ampliprep/COBAS TaqMan HIV-1 Test (Roche Molecular Diagnostics, Pleasanton, CA) and CD4 counts using the Partec CyFlow Counter (Sysmex, Kobe, Japan). Voided urine and rectal swabs were tested for *Neisseria gonorrhoeae* (NG) and *Chlamydia trachomatis* (CT) using the Aptima Combo 2 CT/ NG Assay (Hologic, San Diego, CA). All participants testing positive for HIV or other STIs were offered appropriate treatment regardless of CD4 count.

### Statistical analyses

The primary outcome of this investigation was prevalent HBV infection, and the main explanatory variable was HIV infection status, categorized as infected or uninfected. Potential cofactors evaluated included age (16–20, 20–25, 25–60 years), site (Abuja, Lagos), education (primary, secondary, tertiary), marital status (ever, never, other), willingness to access health care services (yes,no), HBV vaccination status (yes, no), sexual orientation (homosexual or bisexual), sexual positioning (insertive only or any receptive), ever had anal sex without a condom (yes, no), condom use during last anal sex (yes, no), concurrent sexual partners (yes, no), prevalent STIs (any urethral or rectal NG or CT), and vaginal sex in the last year (yes, no).

Distributions of demographic characteristics were compared by HBV status using Pearson’s Chi-square and Fisher’s exact tests for categorical variables. Wilcoxon rank sum test was used to compare continuous variables. Bivariate and multivariable logistic regression models were used to estimate odds ratios (ORs) and 95% confidence intervals (CIs) for the association between HIV and other factors with HBV. Confounders were identified based on biological plausibility, statistical associations with exposure and outcome (*p* < 0.10), and at least a 10% change in the crude beta estimates of the main independent variable. Potential confounders were entered into the multivariable model via forward stepwise selection and retained based on signficance after likelihood ratio testing [28]. Missing observations accounted for less than 4% of the total sample and were retained in the models with indicators. A *p*-value less than 0.05 was considered statistically significant and a p-value less than 0.10 was suggestive of a trend. Characteristics of individuals who were included in the sample versus excluded because of incomplete data were compared using Pearson’s Chi-square test. Data analyses were performed using SAS version 9.4 (SAS Institute, Cary, NC USA).

## Results

Of the 771 participants enrolled from March 2013 to June 2015, 717 had real-time HIV and retrospective HBV testing results available and were included in our study. Out of these, 438 (61%) were HIV-infected and 69 (10%) were HBV-infected, including 41 (6%) with HIV-HBV co-infection. HIV-HBV co-infected participants had a lower median CD4 T cell counts of 345 (interquartile range [IQR]: 163–363) cells/mm^3^ than HIV mono-infected participants, 425 (IQR: 284–541) cells/mm^3^ (*p* = 0.03). Similarly, HIV-HBV co-infected participants had a higher median HIV RNA viral load of 4.7 (IQR: 3.9–5.4) log_10_copies/mL than HIV mono-infected participants, 4.2 (IQR: 2.3–4.9) log_10_copies/mL (*p* < 0.01). Among participants living with HIV, 51% (223/438) had been told by a physician or healthcare provider before enrolling in TRUST/RV368 that they needed to begin treatment for HIV. Of these participants, 24% (107/438) self-reported receiving medication to treat their HIV from a healthcare facility or pharmacy, but not all were able to recall the specific medication regimen they received prior to enrollment.

The median age of participants included in these analyses was 25 years with an IQR of 22 to 27 years. Sixty-two percent of participants identified as bisexual and 75% engaged in any receptive sex (Table 1). Eighty-seven percent had never married, and 93% had at least a secondary school (high school equivalent) education. Regarding health services, 63% were willing to access care but only 5% self-reported HBV vaccination. Compared to HIV-uninfected persons, HIV-infected persons were more likely to be older, married, and engaged in any receptive sex. However, HIV-infected MSM and TGW were less willing to access health care services. The prevalence of HBV in the study population did not differ between those living with or without HIV (9% [95% CI 6.6, 12.1] vs. 10% [95% CI 6.5, 13.6], respectively, *p* = 0.8). After adjusting for potential confounders, HIV was not associated with HBV prevalence (adjusted OR: 1.0, 95% CI: 0.7, 1.6) (Table 2).

**Table 1:** Demographic, behavioral and clinical characteristics of Nigerian MSM and TGW with and without Hepatitis B virus

	HBV+ ***N*** = 69 n (%)	HBV- ***N*** = 648 n (%)	P*
HIV			0.77
Uninfected	28 (10)	251 (90)	
Infected	41 (9)	397 (91)	
Age (Years)			0.51
16–20	12 (13)	81 (87)	
20–25	32 (9)	310 (91)	
25–60	25 (9)	257(91)	
Site			0.19
Abuja	49 (11)	408 (89)	
Lagos	20 (8)	240 (92)	
Education^a^			0.09
Primary	7 (15)	40 (85)	
Secondary	44 (11)	373 (89)	
Tertiary	16 (6)	231 (94)	
Marital status^a^			0.10
Never married	56 (9)	567 (91)	
Ever married	9 (12)	65 (88)	
Other	4 (24)	13 (76)	
Willingness to access healthcare services^a^			
No	24 (9)	238 (91)	0.74
Yes	45 (10)	408 (90)	
HBV vaccination^a^			0.14
No	65 (10)	589 (90)	
Yes	1 (3)	37 (97)	
Sexual orientation^a^			0.08
Homosexual	31 (12)	224 (88)	
Bisexual	37 (8)	421(92)	
Sexual position^a^			0.83
Any receptive	49 (9)	470 (91)	
Only insertive	17 (10)	153 (90)	
Ever had condomless anal sex^a^			0.21
No	5 (6)	81 (94)	
Yes	63 (10)	561 (87)	
Condom use during last sex^a^			**< 0.01**
Yes	33 (7)	426 (93)	
No	36 (14)	216 (86)	
Concurrent sexual partners^a^			0.98
No	19 (10)	177 (90)	
Yes	50 (10)	469 (90)	
Prevalent urethral and/or rectal *Neisseria gonorrhoeae* or *Chlamydia trachomatis*			
No	51 (11)	433 (89)	0.23
Yes	18 (8)	215 (92)	
Vaginal sex partners in past year^a^			
No	21 (13)	141 (87)	0.11
Yes	48 (9)	500 (91)	

*Abbreviations*: *MSM* men who have sex with men, *TGW* transgender women, *p p*-value, *HBV* hepatitis B virus

^a^ ‘n’ may not add up to ‘N’ because of missing values

*Pearson’s Chi-square and Fisher’s exact tests. **Bolded** indicate *p* < 0.05

**Table 2:** Multivariable analysis of HIV and factors associated with HBV in Nigerian MSM &TGW

	Unadjusted OR (95% CI)	Adjusted OR^**a**^ (95% CI)
HIV		
uninfected	Ref	Ref
infected	0.93 (0.56–1.54)	0.95 (0.65–1.59)
Age (Years)		
16–20	1.52 (0.73–3.17)	
20–25	1.06 (0.61–1.83)	
25–60	Ref	
Site		
Abuja	1.44 (0.84–2.48)	
Lagos	Ref	
Education		
Primary	Ref	
Secondary	0.67 (0.28–1.60)	
Tertiary	0.40 (0.15–1.02)	
Marital status		
Never Married	Ref	
Ever Married	1.40 (0.66–2.97)	
Other	3.18 (0.98–9.88)	
Willingness to access health services		
No	Ref	
Yes	0.94 (0.54–1.54)	
HBV vaccination		
No	Ref	
Yes	0.25 (0.03–1.82)	
Sexual orientation		
Homosexual	1.58 (0.95–2.60)	1.53 (0.92–2.55)
Bisexual	Ref	Ref
Sexual Position		
Any receptive	0.94 (0.52–1.68)	
Only Insertive	Ref	
Ever had condomless anal sex		
No	Ref	
Yes	1.82 (0.71–4.66)	
Condom use at last anal sex		
Yes	Ref	Ref
No	**2.15 (1.30–3.55)**	**2.18 (1.31–3.60)**
Concurrent sexual partners		
No	Ref	
Yes	0.99 (0.57–1.73)	
Prevalent urethral and/or rectal NG or CT		
No	Ref	
Yes	0.71 (0.40–1.24)	
Vaginal sex partners in past year		
No	Ref	
Yes	0.65 (0.37–1.11)	

*Abbreviations*: *MSM* men who have sex with men, *TGW* transgender women, *Ref* reference, *NG Neisseria gonorrhoeae*, *CT Chlamydia trachomatis*

Logistic regression was used to estimate odds ratios and 95% confidence intervals. **Bolded** indicates significance at *p*-value< 0.05

^a^The final model was adjusted for condom use during last anal sex and sexual orientation

Those who had not used a condom during last anal sex were twice as likely to be infected with HBV (OR: 2.2, 95% CI: 1.3, 3.6) as compared to those who had used a condom in the unadjusted analysis (Table 2). In the multivariable analysis, condomless sex at last anal sex remained an independent factor and was associated with a two-fold increased odds of HBV infection as compared to those who used condoms (adjusted OR: 2.2, 95% CI: 1.3, 3.6). Although not significant, self-reported homosexual orientation independently trended towards an association with HBV infection (adjusted OR: 1.5, 95% CI: 0.9–2.6). Age, marital status, willingness to access healthcare services, HBV vaccination, sexual position, ever condomless sex, concurrent sexual partners, prevalent bacterial STIs, and vaginal sex in the last year were not associated with HBV. After comparing those included and excluded from the analyses, the retained individuals were less willing to access health care services and more likely to self-identify as homosexual as compared to bisexual (all *p* < 0.05).

## Discussion

The HBV prevalence of 10% observed among MSM and TGW in our study was comparable to those reported by cross-sectional studies of MSM in Lagos, Nigeria (10%) [24] and Brazil (11%) [29] but higher than Tanzania (5%) [30]. Our estimates were slightly higher than previous estimates of PLWH (4 to 8%) in Nigeria with unknown sexual orientation [21–23]. However, our results were lower than those from a study conducted in Ibadan (Southwest Nigeria) where the reported HBV prevalence among MSM was 18% [24]. The higher estimates from the Ibadan study [24] may be attributed to an older age, smaller sample size, and overestimation from enzyme-linked immunosorbent assay based methods commonly used in resource-limited settings [6].

In contrast to our hypothesis, the prevalence of HBV was not higher among MSM and TGW living with HIV. This finding is consistent with a prior systematic review that reported an 8% pooled prevalence of HBsAg that did not differ by HIV among a population of Malawian adults (OR:1.2, 95% CI: 0.8, 1.6) [31]. A small proportion of our participants self-reported taking doctor or pharmacy prescribed medicine for their HIV infection before study entry. If our participants were prescribed antiretroviral therapy (ART) with anti-HBV activity, this may have reduced HBV infectivity [32, 33]. Multiple studies have shown that tenofovir disoproxil fumarate (TDF) containing ART regimen, a first-line regimen in Nigeria [34], have significant anti-HBV activity [35–37]. A systematic review and network meta-analysis of randomized controlled trials [36] confirmed that TDF and tenofovir alafenamide fumarate (TAF) are the most effective ART regimens for HBV virologic suppression. In the Multicenter AIDS Cohort Study, MSM receiving effective ART (HIV RNA level < 400 copies/mL) had the same risk for incident HBV infection as MSM not living with HIV [32]. This pre-exposure prophylaxis effect of TDF containing regimens for HBV co-infections was further confirmed in the Swiss HIV Cohort where there was a reduction of HBV incidence among those on effective ART for their HIV infections [33]. TDF containing regimens may have virally suppressed HBV and prevented transmission for those living with HIV in our cohort.

Although our study did not find a significant association between HIV and HBV prevalence, it offers some insight into how the use of condoms and sexual activities may affect HBV infection. In our TRUST/RV368 cohort, condom usage has been suboptimal and this is a population known to have a high burden of human immunodeficiency virus [38], anorectal STIs [26], and sexual risk practices [39]. Consistent with our findings, the Swiss HIV Cohort study reported condomless sex increased the hazard of HBV acquisition 2-fold among those living with HIV independent of TDF-containing regimens [33]. In Nigeria, Adebajo et al. found an increased odds of HBV infection in Nigeria among MSM engaging in condomless sex that was predominantly for the insertive as opposed to receptive sexual position [24]. In our study, sexual positioning was not associated with HBV infection. Also in the study by Adebajo et al., participants who identified as bisexual were significantly more likely to have HBV (OR:5.2, 95 CI: 3.04, 9.0) [24], whereas our findings suggested the opposite, demonstrating a 2-fold increased prevalence of HBV for those who self-identified as homosexual. Socio-cultural differences in reporting of sexual practices between the North-central (Abuja) and Southwestern regions (Ibadan and Lagos) of Nigeria may affect how participants were categorized [40]. Despite sexual position or sexual orientation, culturally acceptable methods to communicate the benefits of condoms during anal sex for unvaccinated individuals could reduce the risk of HBV and associated morbidities such as ESLD and HCC.

Self-reported vaccination coverage among MSM and TGW was 5% and insufficient in preventing active HBV infection [5]. Routine childhood HBV vaccination in Nigeria was only enacted 15 years ago as part of the expanded immunization program and possibly explains the low vaccination coverage [41]. Most high-income countries with effective vaccination programs have much lower estimates of active HBV. A nationwide, multi-center prospective German study of HIV-infected MSM reported active HBV prevalence as 2%, cleared HBV as 27% and occult HBV as 8% [42]. Similarly, a Danish study from a large tertiary hospital evaluating HBV infection and vaccine coverage among MSM reported active HBV infection as 1%, cleared infection as 7%, and HBV vaccinated as 14% [43]. These studies highlight the reduction in active HBV infection that is possible if a catch-up vaccination strategy were implemented for at-risk individuals in low and middle-income countries.

Our study had some limitations. With relatively small prevalence estimates, the multivariable model was not sufficiently powered to detect independent associations between HBV infection and known demographic and behavioral factors. However, most associations between sexual behaviors such as homosexual orientation and exclusively male partners with HBV infection aligned with the direction of the study hypothesis. Although, because more MSM who self-identified as homosexual were retained in our analyses, our prevalence estimates may have been an overestimate because of enrichment of those engaging in high risk sexual practices [38]. HBV vaccination was self-reported as ever receiving and did not specify the number of doses. Additionally, it was unclear who had been exposed to HBV from vaccination versus a prior infection, as testing for HBV surface antibody was unavailable due to limited sample volume. Due to uncertainty regarding participants’ past exposure to specific ART regimens, we were unable to assess whether HBV prevalence was lower among those living with HIV and exposed to TDF-containing regimens. The temporality of associations between HIV and HBV could not be determined because of the cross-sectional study design. Lastly, MSM and TGW originated from two cities in Nigeria and results from this study may not be generalizable to other regions of Nigeria.

## Conclusions

HBV prevalence was moderately high and comparable to other studies of MSM but did not differ by HIV status. Recent condomless anal intercourse elevated the risk of HBV, reinforcing the need to increase communication and education on HBV and its potential long-term complications. Further studies are needed to confirm whether the attenuated HBV prevalence among MSM and TGW living with HIV was the result of TDF-containing ART regimens with anti-HBV activity.

## Data Availability

In order to protect the clinic staff and the participants from stigma and criminalization of same sex behavior, the data has not been made publicly available. The data are available from the corresponding author on request.

## Abbreviations

HBV: Hepatitis B Virus
MSM: Men who have sex with men
TGW: Transgender women
OR: Odds ratio
Cis: Confidence intervals
WHO: World Health Organization
ELSD: End stage liver diseases
HCC: Hepatocellular carcinoma
HbsAG: Hepatitis B surface antigen
PCR: Polymerase chain reaction
PLWHs: Persons living with HIV
RDS: Respondent driven sampling
STI: Sexually transmitted infections
NG: *Neisseria gonorrhoeae*
CT: *Chlamydia trachomatis*
IQR: Interquartile range
ART: antiretroviral therapy
TDF: Tenofovir Disoproxil Fumarate
TAF: Tenofovir Alafenamide Fumarate
DNA: deoxyribonucleic acid
RNA: Ribonucleic acid
